# Elderly Care: A Study on Community Care Services in Sleman, DIY, Indonesia

**DOI:** 10.1155/2020/3983290

**Published:** 2020-05-07

**Authors:** Sumini, Sukamdi, Evita Hanie Pangaribowo, Yeremias T. Keban, Muhadjir Darwin

**Affiliations:** ^1^Doctoral Program in Leadership and Policy Innovation, Graduate School, Universitas Gadjah Mada, Yogyakarta 55281, Indonesia; ^2^Center for Population and Policy Studies, Universitas Gadjah Mada, Yogyakarta 55281, Indonesia; ^3^Faculty of Geography, Universitas Gadjah Mada, Yogyakarta 55281, Indonesia; ^4^Faculty of Social and Political Science, Universitas Gadjah Mada, Yogyakarta 55281, Indonesia

## Abstract

Elderly care services are important to provide in response to the rapid growth of the elderly population. In developing countries like Indonesia, the speed of growth of the elderly population does not simultaneously occur, so the needs for care services vary. This study discusses the emergence of home care services in response to the increase in elderly population. By taking the case of community home care services in Sleman, this study found the pattern and process of the emergence of local initiatives in home care services. This study also revealed an important factor affecting the implementation of community home care services, that is, leadership.

## 1. Background

The increase in ageing population has become a global phenomenon [1]. Most developing countries have already had this special achievement [2], including Southeast Asian countries, such as Indonesia. Aging population in Indonesia increased from 4.5 percent in 1971 to 9.6 percent in 2019 [3] and is thought to be a trigger for the widespread of the community care for elderly [4]. Anxieties over the problems from the increasing number in elderly have encouraged social movements and initiatives in the community. Situmorang [5] said that community initiatives and social movements in Indonesia are related to the failure of the government system to guarantee welfare. This is evidenced by the state budget of around 2 percent per year allocated for social welfare, lower than that of Vietnam and Thailand which is around 5 percent per year [6]. As the implication, the government's ability to provide elderly care is very limited. The government has only been able to provide nursing homes spread over one unit in each district/city [3] to date and this is not sufficient to accommodate the growing elderly population.

Lehning, Scharlach, and Wolf [7] revealed the importance of studies on local initiatives in elderly care practices. In addition to describing social processes in the community, local initiatives also help to find an elderly care model to complement the long-term care policy for the elderly, including supporting active aging policies [8]. Community care initiation for the elderly, such as home care, has also been proven to improve the elderly's quality of life. The study of d'Orsi and Jette [9] showed that the quality of life of the elderly is related to sociodemographic factors, such as social relations. The interaction between the elderly and their social environment will prevent them from the risk of social vulnerability. Home care services make it possible for the elderly to continue interacting with the surrounding environment and avoid the risk of feeling lonely [9]. However, studies that discuss the initiation, form, and product of home care services by the community, substance, and community resource constraints have not been widely conducted [10]. This is in line with criticism made by Coulter et al. [11] that the studies of elderly care need to look at the aspects beyond general care (i.e., from the quality of service practices performed by formal government and private institutions to community initiatives). In line with the criticism of Davitt et al. and Coulter et al., this article intends to discuss the practice of elderly care that is initiated and organized by the community.

## 2. Introduction

The family program, as a population control policy which was implemented in 1970, has produced different demographic changes without being coincided by the Government of Indonesia. Five regions, such as DIY (Yogyakarta Special Province), East Java, Bali, Central Java, and South Sulawesi, are known to enter the population ageing earlier. This condition is in line with Hugo's prediction [12] that DIY will reach the aging structure earlier. He claims that the speed of the increase in elderly population in DIY is related to a decrease in birth rates. In fact, DIY has reached replacement level marked by a TFR of 1.8 in 2010 and 2.2 in 2017, resulting in an elderly population of around 14 percent in 2019 and has become the highest rate in Indonesia [3, 13]. The percentage is expected to increase and reach 20 percent in 2025.

The increasing trend of the elderly population in DIY is expected to trigger an increase in the need for homecare services [12]. Studies that specifically estimate the level of need or adequacy of homecare services have not been well documented. This might be related to the speed of achieving different aging population in each region in Indonesia, so the studies have not yet been directed towards the elderly. Studies that specifically discuss the practice of community care are rarely found. Studies on elderly care in Indonesia are mostly conducted in a small scale [14–16] and tend to lead to health due to the decrease in physical organ function [17], mental [15], and elderly care performed by families [18–20], whereas the condition of 66 percent of the elderly in DIY, which is very dependent on families whose role is currently declining, had made it crucial to fulfil the need for assistance of everyday activities and mobilities [4]. There are no specific and exact data on the number and distribution of community home care practices in Indonesia, but such practices can be found in some areas which have reached and entered the elderly population stucture, such as in DIY [13].

Correspondingly, community care for elderly was found in one district in DIY, that is, Sleman [13]. Its existence is important to find out due to its role in supporting the lives of the elderly; that is, around 50.38 percent of them needed an assistance, complementing the services of government nursing homes and social institutions that provide homes for the elderly [21]. Community care for the elderly in Sleman does not bring the elderly to nursing homes or new homes, but it provides services at home through home care. This is in line with the results of the study which indicate that the elderly prefers to live in a familiar environment [22, 23]. Community care for the elderly in Sleman that was established by local initiation has the opportunity to grow, being a development model for other areas that are experiencing population ageing. This study is crucial because it provides an overview of the role and involvement of the community in elderly care practiced in developing countries, which has been assessed by Gupta [24] as not yet receiving serious attention up to now.

## 3. Health and Social Protection for the Elderly in Indonesia

A policy on the elderly in Indonesia is regulated in Law Number 13 of 1998. One of the important things is that of regarding elderly services. The government provides different services according to the elderly category which are grouped into two: potential and nonpotential elderly. Potential elderly is those who are still economically and socially productive, while nonpotential elderly is those who are powerless, unable to carry out any economic activity, and highly dependent on others. Training, education, and job opportunity services are provided to potential elderly, while social protection services are provided to nonpotential elderly because they are considered not to have the ability to do any economic activity.

The present law has become a lot of public security since it is considered not sufficiently relevant to solve the challenges and problems of the elderly, so reviewing is a recommended choice [25]. Providing services to potential elderly that only cover education and training, employment opportunities need to be expanded to guarantee employment, while nonpotential elderly services need to pay attention to those who suffer from chronic illnesses, Alzheimer, dementia, and complex diseases because they require long-term care. Although being often considered to be less “up-to-date” with current conditions [26], this law remains a reference for the provision of elderly services, such as the following services:

1. Nursing home services which are managed by the government: in this case, the government provides services in the form of housing for elderly who are neglected or do not have families. It covers the need of clothing, food, social, religion, and recreational and health checks by medical personnel on a regular basis.
2. Nursing home care managed by nonprofit institutions: the services are not much different from government nursing homes. The difference is that it is not limited only to the neglected elderly and those are without family, but to all the elderly. However, the term “nursing home” has been embedded in Indonesian society as a place to live for elderly who are poor, are neglected, and do not have families, so it shows unfavourable stigma. It also develops cultural and religious values in the community to elderly care as a form of respect for their merits. Giving up parental care affairs to the nursing homes is seen as neglect. The management of this service is very dependent on donations from government and social institutions, the community, and foundation.
3. “Senior living” services managed by the private sector: it has begun to develop in recent years. Private sectors use positive term, such as the term “senior living” rather than the term “home.” To be able to access this service, people must be willing to pay a relatively expensive fee. One of the examples is the senior living cost in the Capital City of Jakarta that reaches IDR 16 million per month. With an average income of the Indonesia population per capita that is generally around IDR 2 million per month, it is only upper-class people who can access it.

**Figure 1:**
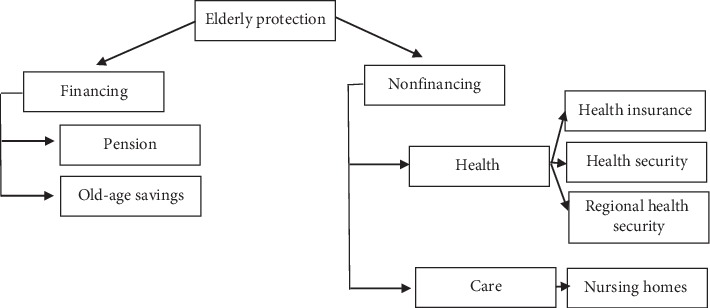
Elderly social protection scheme in Indonesia (source: Bappenas, 2015).

Meanwhile, social protection for nonpotential elderly is specifically regulated in Law Number 40 of 2004 concerning the National Social Security System. In its implementation, as presented in Figure 1, elderly social protection policies are grouped into two: financing protection and nonfinancing protection [13]. The former is provided through future savings and pensions. This scheme only reaches elderly who retire from civil servant status (including the police and military officers). The latter is given when they start to retire. These funds are the result of routine deductions of income per month during the work period, while old-age savings are the money provided by the government at 60 percent of the income when actively working. The savings are given during retirement, meaning that the elderly with civil servant retirement status do not need to worry about their retirement because the government guarantees their finances until the end of their lives. The retirement age is stipulated on 58 years for employees, 65 years for lecturers, and 70 years for professors.

For those who work in the private sectors, the government implements a pension policy that is charged to each company. The mechanism is the same as that of civil servants, where the company deducts part of the employees' income in preparation for retirement. During the period of employment, the funds will be accumulated and provided at the end of their tenure. The pension funds can be used as economic provisions to begin the retirement. Even though the reality is not the case, many retirees use it to buy cars and go for Umrah or Hajj [16].

The elderly who work in informal sectors get financing protection through social assistance, such as elderly social security managed by the social ministry. The social security provided to the elderly of IDR 300 thousand per month has been held in stages since 2006 in Yogyakarta, Jakarta, Banten, West Java, Central Java, and East Java. The scope continued to be expanded until 2010, reaching 29 provinces out of 34 existing provinces. This social security is prioritized to nonretired civil servant elderly aged 65 years or 70 years or over with physical conditions that make it impossible to work among the poor [26].

Nonfinancing protection covers health and care protection. Health protection for the elderly consists of health security, BPJS health insurance, and regional health insurance. Health insurance is given to elderly with civil servant retirement status, while BPJS is an insurance system provided by the Government of Indonesia for all people. Families are required to register themselves in this health insurance by paying contributions per month per person in accordance with the provisions. In addition, the community and the elderly can also access regional health security through submissions to the regional government, especially those from families with low welfare level.

## 4. Object and Method

### 4.1. Object

This study took the case of community home care services in Sleman, DIY, Indonesia. The choice was based on two important reasons. First, community home care services in Sleman, DIY, is the first service in Indonesia that emerged from community initiation [13]. Second, community home care services have the opportunity to be developed in other areas that will later enter population ageing, so an analysis of the initiation and management of community home care is important to find out.

### 4.2. Method

This study applied a qualitative method with an emergent design intended to allow the study to be undertaken from a researcher's perspective. The study began with a series of in-depth interviews with an informant who had initiated home care service. Through snowball sampling (i.e., informant recommendations), further informants were identified, including home care providers (popularly known as companions), head of village, head of hamlet (community units consisting of four neighborhoods), and head of neighborhood (persons responsible for 25–45 families). These informants were approached directly and then interviewed by the researcher. Further data were collected through telephone interviews with healthcare partners, including medical staff involved in house of care services. As such informants were highly active and mobile, often travelling from village to village, it would have been difficult to schedule time for direct interviews.

Interviews with informants began with the collection of demographic information, including age, marital status, educational background, and employment. Interviews continued with questions about house of care services, house of care mechanisms, and the reasons for house of care. During interviews, the researcher took notes about informants' actions with their cellular phones, the sounds of passing motor vehicles, and informants' responses to questions. These notes helped the researcher probe for further detail or ask for clarification. The collected data were processed through grouping and categorization. For a more comprehensive analysis, this study was also supported by secondary data obtained from health reports of the elderly.

## 5. Results

The results are separated into three sections. The initiation process and resource mobilization were examined in the first section. The management of home care services was examined in the next section and followed by the last section, namely, the community involvement.

### 5.1. Initiation and Resources Mobilization

The initiation for providing elderly home care services was first established by a community member named Sudirman (not his real name). He is a graduate from education program, works as a civil servant in the village administration, and has a wife who is a midwife. He has good experience in taking care of his mother who suffered from a stroke disease. He had to take care of his mother for ten years, who lost all her motoric functions, was paralyzed, and could only lay on bed.

“I had to take care of elderly for years who suffered from stroke disease. Nobody knew what it was, like to live for years without being able to do anything, just because she couldn't protest, so we just left her, like it's not what a humanity look like, aren't we? But I took care of her, bathed her, fed her, until the end of her lives.”

Such condition was an important turning point in his life. He was encouraged to help the elderly around his residence in order to get the better care. According to him, not all elderly around him could live well, live in well-off households, and have old-age saving. There were also those who still had to work in the field and when they were sick, they could not look for the treatment because they did not have much money and had increasingly severe symptoms, such as hypertension, headache, and acute infection of respiratory. Based on the records from the local health office, such disease is commonly undergone by the elderly as presented in Table 1. Even though there was a health assistance scheme from the local government, some elderly did not know how to access it.

Hypertension, as a disease that is often undergone by the elderly in Sleman, is believed to be the cause of stroke disease. In the case of stroke disease, Sudirman observed that there is a need for long-term care. At severe pain like being unable to move or paralyzed, the examination should be done at home. Similarly, for the elderly who suffer from various complex diseases, home care becomes an important requirement. Some hospitals in Sleman have provided home care services, but not all elderly families have the ability to pay it. Moreover, if the home care is long-term services, many elderly families cannot afford it, so caring for elderly in a makeshift home until the end of their lives will finally be the last choice.

“Elderly families here, sorry to say, can't pay for the treatment and care because they're still lacking in fulfilling the needs for daily food costs, especially to request home medical personnel. It is no wonder that the elderly is cared for perfunctory, given food, bathed, yeah just like that. If there are complaints, such as pain or dizzy, they will be smeared with eucalyptus oil”

Sudirman initiated a visit to elderly homes around his residence. He initially encouraged his close friend who is a Posyandu cadre (Posyandu is an integrated service post that was developed in the 1970s to monitor the health and development of infants and toddlers. It is held every month by providing service, such as the measurements of weight, head circumference, and height of infants and toddlers. The service is then extended to the elderly, such as measurements of blood pressure, body weight, and supplementary feeding. In carrying out this service, the Posyandu is supported by community members who are referred to as Posyandu cadres, the members of community chosen on the basis of their activity, willingness of time, and commitment to do Posyandu social works). The visit was only intended to greet the elderly and the activities included asking the elderly's health condition, the pain, listening to the past experiences, and the daily routines of the elderly. The visit lasted about 30 to 60 minutes and was performed to one to two elderlies per day.

The visit continued to be performed despite getting unpleasant responses from the community members from the surrounding people who were invited to participate in caring for the elderly as Sudirman recalled as follows:

“It's useless, just visiting their houses. They have children and families after all, so they could have taken care of them. Don't be a brown noser!”

“If we want to find the merits, we shouldn't show it off. They (the elderly) don't need it [attention], especially by other people, even it can offend their family”

Based on the visits, it reveals a description of the problems faced by the elderly. The problems include difficulties in doing daily activities such as eating, drinking, bathing, urinating, and loneliness because there were no friends to talk, limited access to entertainment media, inability to buy additional foods other than staple foods, disturbances, and complaints sick as well as the lack of clean places to live. The problems that were identified after approximately three months of regular visits to the houses lead to the next stage, which is to design the activities performed at each visit. The visit was initially only a greeting which was then designed with special contents at each visit.

Sudirman divided elderly care into four service activities; (1) personal care, (2) accompanying care, (3) household care, and (4) health care and referrals. Personal care is a care provided to help fulfilling the personal needs of the elderly, such as eating, drinking, bathing, hair washing, tooth brushing, and clothes changing. Accompanying care focuses on the social bonds formed between the elderly and their companions. The aim is to foster the self-confidence of the elderly that they are happy individuals and do not live alone. The care is provided in the form of accompanying the elderly, either listening to the stories or reading stories to the elderly, accompanying them watching television, and accompanying them walking around their residential area. Household care is given to the elderly related to household matters. In this case, the companions give a support in the form of cleaning and changing bed linen, folding and washing dirty clothes, and cleaning the rooms. In addition to these three services, visits to elderly homes are also provided with health care and referrals. Health care aims to determine the health condition of the elderly. It is important to determine the actions that should be taken if the elderly's health is getting worse, including the referral to secondary health services at further level. In terms of health care, Sudirman asked his wife to work. She is very supportive of Sudirman's efforts and she helped checking the health of the elderly at home, such as checking blood pressure and pulse, body temperature, and cleaning the body of the elderly who suffered from paralysis and laid in bed.

Along with visiting to the elderly house that Sudirman has done for two years (2002–2004), he also continued to try mobilizing the community to be involved in caring for the elderly. At every opportunity of the community meeting, he brought a message about the problems faced by the elderly and called for community involvement. He realized that the community is an important asset and resources to support the running of home care. He also did not forget to contact the figures in his environment for the expansion of ideas, such as the head of hamlet, head of village, religious leaders, and head of RT. This indicates that the change he has initiated was disseminated to various elements. A process that was carried out repeatedly and required relatively long time had showed significant results. He was able to establish the same view among the community, leader, and village government that the elderly needed social support. It is that kind of common vision that becomes the resources and asset to move together in providing free home care services.

The home care service was officially established in 2004 under the coordination of Sudirman. He received support from the village government and community, so some activities such as meetings and discussions were held at the village office. The support also came from the head of hamlet and head of community whose orders were obeyed by the people. This has implications for the number of volunteers who registered and the increasing demand for home care services. There were 10 volunteers and the elderly who had been served are all the elderly of the village. Previously, Sudirman went from one home to another that was considered to be potential volunteers to get the support. Not all of these potential volunteers were willing, especially since they did not necessarily have the required funds, time, or energy. The reasons are as follows:

“many refused … they wouldn't receive money, so they preferred dealing with their own households or collecting feed for their cattle. I could accept that. I really understood” (Sudirman).

“Of course, there were those who refused. It was social work, and unpaid, so if they didn't feel drawn to the issue, why would they do it? Taking care of the elderly … it was hard, and taking care of the elderly is much different from taking care of babies. I was reminded of my parents and the fact that I would be elderly one day” (Volunteer).

### 5.2. Service Management

The implementation of home care services is very dependent on funding and assistance from other parties. This is one of the obstacles faced by community home care. Sudirman was aware of these challenges and sought alternatives in two ways, such as (1) developing collaboration with stakeholders and (2) encouraging active participation from the community.

The collaboration was begun by mapping the stakeholders associated with community home care services. Several stakeholders have been identified at present, including the village government, midwives, puskesmas, and academics. It was formally built through the submission of proposal to the related institutions. The collaboration with the village government had benefited the development of community home care. The village government of Sendangadi-Mlati, Sleman, provided a room in village office for the secretariat. To support the day-to-day operation of the secretariat, the village government also assigned additional duties to some employees to assist with administrative matters, such as correspondence (letters writing) needed in community home care services. They also allocated annual funds to support the community home care services. The amount varies each year. It depends on the village government's income. The funds are used to support the delivery of home care visits, such as buying food and oil gift for the elderly.

This study also found that the collaboration with village government has made community home care become more established. The government support requires that community home care services can officially be registered as a social organization. It means that community home care services must be registered with a government social institution and have been certified or permitted to operate in the community. The conditions needed to facilitate control of the use of funds from the village government turned out to have implications for the need for organizational structure. The community home care initially ran under Sudirman's coordination, but then it already has a clear organizational structure.

The collaboration with medical services, that is puskesmas, was performed to obtain medical support for home care visits. Although the puskesmas was willing and supporting community home care services, not every home care visit was always assisted by the medical personnel. This was admitted by Sudirman due to the limited resources at the puskesmas which also had to provide health services. Sudirman said that, after the puskesmas personnel being involved, the local community became more supportive of the home care and began recommending potential service recipients. This support is also extended to service provision, as more people became the volunteers and the volunteers have reached 40 persons. This, on the one hand, proved beneficial, as it has enabled more services to be provided. On the other hand, it has posed a challenge, as volunteers' different backgrounds, knowledge, and skills that affect their individual abilities to provide home care services. The condition has pushed for the development of collaboration with academics, such as geriatricians and psychologists. They were invited to a training held by community home care to practice skills and increase understanding on elderly care.

The home care training was chosen as the best means of improving volunteers' skills and standardizing their knowledge of home care techniques and methods. An average of two or three training sessions has been held per annum, with materials catered to volunteers' needs. For example, some volunteers had to deal with elderly who suffered from Alzheimer, and as such they requested a special session on treating patients with Alzheimer. Similarly, volunteers noted the devastation that earthquakes could cause among elderly and thus sought psychological training.

“All of us. My colleagues and I designed the proposals. For example, in the field we had to deal with people with Alzheimer's … we'd have already bathed them, and they'd say they hadn't bathed. We'd have already fed them, and they'd say they hadn't eaten… always, always like that. At the time, we weren't ready for such things. When we gathered and discussed the matter, we decided we needed training, needed to learn about Alzheimer's, and so we decided to hold a training session. Similarly, when the earthquake happened a while back, we decided we needed help with the psychology of the matter, and so we held a training session and invited a psychologist.” (Sudirman).

The main source of training fund came from the volunteers. They used their own money based on the agreement to make the training successful run. Their money was used to pay all related costs, including speaker fees, materials, and snacks. They did not mind the expense, as they perceived the training as necessary for improving the quality of their home care services.

“That's right … they [volunteers] paid and they didn't mind. We got some knowledge … how could you get knowledge without paying? We knew that we needed this knowledge, needed to know how to bathe elderly who can do nothing except lay down, how to bathe them without lifting them and moving them from their beds, how to put a thermometer in their armpits. We didn't know the right way, and so we received training, knowledge, learned how to do it right. And if we had to pay? Well, it's only fair.” (Sudirman).

In addition to developing collaboration, the next way is to encourage community involvement in community home care. The community was encouraged to be involved in community home care according to their abilities. The steps included mapping and identifying the economic potential that exist in the community, especially trade and service. The trade sector included culinary businesses and groceries shops, while the service sector included laundry businesses that were commonly found around the community. They were encouraged to be involved in caring for the elderly by participating through good trading or services. Those who had culinary businesses were expected to take care of the elderly by providing food for the elderly in surrounding area. Also, those who had a laundry business were expected to be able to help the elderly by providing free services. The support mechanism provided by the community had not been regulated yet or there were no specific provisions, but rather social solidarity. This allowed some elderly to get more social and economic support from their environment and some did not. This is a challenge for the community home care going forward to build a more certain system and ensure the community involvement.

“We've worked together with merchants and shops. We said, ‘If the elderly comes to buy household needs like sugar and tea, please let them take it for free.' They know that, for elderly customers, they should not ask for money. Even the travelling meatball venders, if they are in front of a senior's home, they'll give them a bowl of meatballs” (Sudirman).

“We've taught them, with the support of the hamlet and the neighborhood leader. We've explained that our lives must benefit others. We need to share our blessings with others, to make sure we don't impoverish those in need. We need to enrich them, to help them live peacefully and happily. That's what we need to do. People have felt how, after doing good deeds, they have happy hearts. They are joyful.” (Sudirman)

### 5.3. Community Involvement

The willingness of community to get involved in performing home care services without any compensation is not merely related to Sudirman's ability to deliver social messages to them. Moreover, a number of assumptions expressed by Leveritt et. al. [27] and Kørnøv and Thissen [28] show that (1) the community can make the best decision because they have good information, (2) all individuals act rationally and logically in a consistent way, and (3) the way they tend to improve their welfare was found in this study. The volunteers said that they were willing to join home care services because they knew the vision and goals clearly. The information on elderly issues and the needs for home care services had inspired them to directly get involved. In addition, they had also really thought about the implications of their involvement, such as the duty to allocate time and energy without any compensation. This voluntary activity was based on their desire to help and share with others. It was recognized that they were able to present their own satisfaction and happiness when their existence could have given benefits to others. This happiness is one of the important immaterial welfare benchmarks to consider.

“I am willing to be a volunteer because I have time. It'll way useless if my spare time is not used to do positive things, can be useful for others and get merits too.” (Volunteer)

“I knew from the beginning that being a volunteer means that I'm not paid. Mr. Sudirman is as he is (fully explained it) about what we should do. It does not matter to me (not paid), it's a kind of worship.” (Volunteer)

The willingness of the community as volunteers is also related to influence of peer, culture, and social networks [29, 30]. Most volunteers had interrelated social networks, such as Posyandu cadres. The network raised social ties and encouraged one another to engage with other activities, including home care services.

“Here (home care service) I followed my friends (other volunteers), I was invited and interested. I was also used to activities related to elderly care, such as taking care of the elderly every month in Posyandu.”

**Figure 2:**
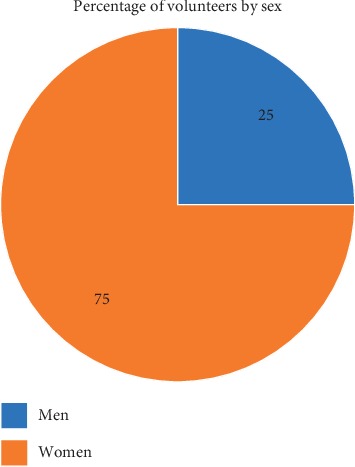
Percentage of the volunteers by sex (source: profile of home care service organizations, 2019).

In addition to the above factors, this study also found patterns that indicate the high involvement of women. As displayed in Figure 2, from the 40 volunteers, 75 percent of them are women. This is inseparable from the function and role of care for elderly that are commonly inherent in women [31]. Meanwhile, to allocate time for daily activities, taking care of the household and performing home care services are none of the problems for them, because the visits are only conducted twice a week with the following details in Table 2. In every visit, the volunteer allocates around 70 minutes. The volunteers have a short session for briefing and takes 30 minutes, while a visit to the elderly takes around 40 minutes.

## 6. Analysis

Home care services grew out of community efforts in Sleman to provide support to elderly who were not receiving proper care from the government. As such, it may be identified as what Reinsberger et al. [32] term a grassroots initiative. These services, as with other grassroots initiatives, are characterized by a bottom-up approach [32], noncommercial orientation, and reliance on local potential and resources [33].

The home care services began as a bottom-up initiative. The idea began with a community member, who then received the support of community members. Bottom-up initiatives are generally unstructured and unorganized, and as such their genealogies are difficult to trace [34]. Nonetheless, this bottom-up approach has been widely adopted, as its community focus enables movements to recognize the needs and issues of their communities and identify appropriate strategies [34, 35]. This can also be seen in community-based elderly care services, which are better able to identify the issues faced by elderly and thereby promote real action.

Such a bottom-up approach also promotes community empowerment [36], as it encourages communities to become involved in elderly care; this can be seen, for example, in merchants' willingness to provide food and food products to elderly at no cost and in neighbors' willingness to help wash elderly' sheets and clothing. Owing to its bottom-up approach, it has been able to continuously provide home care services to elderly and this reflects the common view that bottom-up approaches promote continued innovation [34, 37].

The success of the bottom-up approach depends significantly on social and cultural considerations [38, 39]. Most residents of Sleman continue to adhere to traditional sociocultural values, including tolerance, helpfulness, and togetherness. This can be seen, for example, in members' willingness to provide care to elderly, so the home care could not possibly succeed without such sociocultural values.

Elderly home care services in Sleman are not business oriented, nor do they follow the top-down approach of commercial entities [40]. Volunteers attend to elderly free of charge, receiving no financial compensation for their personal, household, social, or health care services. Activities are funded through donations, grants, and volunteers' own contributions; these have been supplemented in recent years by funds from the village government. This reflects the findings of Feola and Nunes, who argue that successful grassroots movements draw on donations, grants, and the support of local governments. Funding is determined by donor institutions' concern for and commitment to an issue, as well as the concern and commitment of the government [29].

The home care services have also relied on local resources which are needed for successful innovation. Despite several limitations, it has not stopped providing continuous elderly care. Rather, it has expanded, drawing on community members' support and resources to provide a broader range of services.

This study has also underscored the importance of leadership. In bottom-up movements, the leader has a very important role. There must be someone who comes up with ideas, who convinces others to become involved, and who takes charge. There must be someone who, despite never having desired a leadership role, takes control after growing dissatisfied with the social reality. There must be someone who has a different view of the situation, someone who sees situations that others have accepted and thinks “there must be a change.”

In the case of the home care services, this leader was Sudirman, who recognized the difficulties being faced by elderly who lived on their own in poverty, without any family support. Even though his community saw this situation as normal, Sudirman drew on his personal experiences and observations. He sought to open their eyes, to show them that they could not let elderly suffer.

“Here, in Sleman (Regency), it's a common thing. Elderly live on their own, abandoned by their children and families, even though elderly have the same rights. The same right to dignity, to happiness, and to decent living. I couldn't stop thinking … what about the elderly? What would happen if they fell, if they became ill, or … I couldn't forget imagine what it was like to just abandon one's grandparents. Elderly living on their own, maybe only two teeth left … there had to be someone ngopeni [taking care of them].

I had to take care of elderly for years … strokes … for years. Nobody knew what it was like to live for years without being able to do anything … just because they couldn't protest, people accepted it. It was inhumane. But I took care of them, bathed them, fed them, until the end of their lives. I didn't imagine that these home care services would become like this. I've had the opportunity to travel to Singapore, to visit other areas … I never imagined things would be like this. I just couldn't imagine letting them go without any care.”

Several elements of fundamental leadership are described as follows:

1. Capacity for change. The home care leader must be capable of recognizing unjust, illegal, and unethical conditions [29], as well as transforming them. This capacity for change requires a high level of social concern and sensitivity. It resembles what Senge, 2006 [41], describes as the capacity to recognize links between components and promote the betterment of society. Homer-Dixon, 2000, in Senge, 2006 [41], described a similar capacity, namely, the ability to recognize the complex socioeconomic issues in society.
2. Involvement in various processes. Home care services in Sleman were initiated by a member of a local community, a person without any formal position or authority. As such, this individual was simultaneously working to support his family, to provide home care, to provide training to volunteers, to obtain funding, to plan activities, and to otherwise realize his goal. The leader, thus, was involved in every aspect of home care, including networking and collaborating. Collaboration is identified as one key to elderly care services [42]. The strategic partnerships between elderly care and all sector including university, local government, community, and healthcare service (in this case represented by Puskesmas) are considered integral to community home care sustainability.
3. Using effective and innovative approaches and strategies. All leaders seeking to create change will face a range of challenges, including limited funding, Middlemiss and Parrish, 2010 [43], and community opposition. A leader must thus remain dedicated to their purpose (such as promoting elderly welfare). In overcoming these challenges, the leader must use effective and innovative approaches and strategies to minimize input and optimize output/results. In the case of home care services, several approaches were used. First, the leader did not require volunteers to implement his own ideas but rather accepted and accommodated their input. He discussed their recommendations with other volunteers, and they agreed upon a solution; this can be seen, for example, in the decision to teach volunteers about Alzheimer. Second, the leader worked to empower the community and obtain their support. He sought to involve community members in accordance with their abilities. Community members provided food, helped doing laundry, and provided elderly with other forms of support. They were not forced to be involved, but rather became interested on their own and contributed of their own volition.

## 7. Limitations

This study has provided a portrait of home care services provided by volunteers in Sleman, DIY, Indonesia, including the forms and initiatives taken. This study has found that the home care services provide personal care, social care, household care, and healthcare/referrals. Although this study recognizes that these care services differ from those provided by formal institutions, it has not conducted any comparison to examine these differences in detail. It has only discussed services in terms of their providers, the initiator, volunteers, and partners. This study has not involved service consumers such as elderly or their families. Similarly, service quality and consumer satisfaction have not been examined.

Within the context of home care service initiatives, this study has found that leaders have a central role. Services initiated at the local level (i.e., grassroots initiatives) can prove innovative, as shown by the case of home care services in Sleman. However, recognizing that much of the literature on grassroots initiatives has focused on sustainable development, energy crises, and natural resources, we must note that subjectivity is still a factor. Studies focusing specifically on elderly house of care services, including the situations, processes, and grassroots initiation and movements, are thus strongly recommended.

This study has yet to identify specific categories and types of leaders based on their involvement in elderly home care services. Although it has analyzed the character of one specific leader, it has not conducted any categorization. As such, further research into elderly home care services should consider the different types and models of leadership. This would fill a serious gap in studies of organizational leadership, particularly in developing countries.

Finally, this study has only focused on one specific area, namely, Sleman Regency, DIY, Indonesia, where a community-based elderly care services have emerged. Given this focus on one specific territory and one specific community, the findings of this study may not necessarily be applicable elsewhere. The sociocultural context of a community informs its ability to successfully innovate. In Sleman Regency, DIY, Indonesia, where values of togetherness and tolerance remain common, the sociocultural context proved beneficial to the social mission of the service providers. Elsewhere, the sociocultural context could be detrimental.

## 8. Conclusion

Community home care services can be developed with proper leadership. The leader must be capable of identifying issues in communities, suggesting changes, taking real action, promoting voluntary involvement, and developing networks. Such leader is necessary to realize breakthroughs and innovations, including strategies for overcoming obstacles and hindrances. In the case of elderly home care, a leader plays an important role to manage the organization and the home care services do not only depend on the initiators but also depend on many factors, such as social and cultural, society responses and their participation, and local government responses. They work together and support each other to perform the home care services.

Elderly home care initiated by local figures may more establish because it emerges from local with bottom-up process, so it can capture the local social problems more accurately. It has worked to identify the adequate services, cover personal care, household care, social care, and health care based on assessment; even the local initiative services tend to have limitation on budget. A leader may work to find a strategy in order to collect funds to support the services.

In the context of replication, good practices of elderly care in Sleman, DIY, is trusted to implement in elsewhere. However, we need to be careful to interpret the role of leadership. A strong character possessed by a leader may need special attention in analyzing process, because it needs more than just a leader, but a complex combination of a leader with intelligent aspects, such as a passion in giving care for elderly, working with heart, having good respect, tolerance, and sympathy to others.

## Figures and Tables

**Table 1:** Types of diseases suffered by the elderly in Sleman, 2017.

Types of disease	Amount	Percentage
Primary hypertension	20,438	14.9
Diabetes mellitus	9,472	6.9
Other disorders of muscle tissue	6,725	4.9
Common cold	5,718	4.2
Dyspepsia	4,998	3.6
Pulp disease	4,078	3.0
Headache	2,866	2.1
Heart failure	2,551	1.9
Joint disorders	2,089	1.5
Other acute infections of the upper respiratory tract	2,057	1.5
Cough	1,984	1.4
Stroke	1,744	1.3
Other arthritides	1,382	1.0
Gastritis	1,320	1.0
Acute pharyngitis	1,208	0.9
Other diseases	68,630	50.0
Total	137,260	100

Source: District Health Office, Sleman 2017.

**Table 2:** Details of home care activities per visit.

Activity	Duration
Briefing	30 minutes
Visit	
(i) Personal care	15 minutes
(ii) Home care	15 minutes
(iii) Health care^*∗*^	5–10 minutes

Source: interviews with the volunteers. ^*∗*^Performed only if there were medical personnel from Puskesmas. Otherwise, it could be done with physical exercises.

## Data Availability

The data used to support the findings of this study are available from the corresponding author upon request.
